# Passing is not entrusting: rethinking intensive care certification in the era of artificial intelligence

**DOI:** 10.62838/jccm-2026-0063

**Published:** 2026-10-30

**Authors:** Alexandru Nica, Gizella Melania Istrate, Pedro Povoa

**Affiliations:** Intensive Care Department, Centre Hospitalier Intercommunal Compiègne-Noyon, Compiègne, France; European Society of Intensive Care Medicine, Brussels, Belgium; NOVA Medical School, CHRC, NOVA University of Lisbon,Portugal; Department of Intensive Care, Hospital de São Francisco Xavier, ULSLO, Lisbon, Portugal; Research Unit of Clinical Epidemiology, Department of Clinical Research, Odense University Hospital,Denmark; D’Or Institute for Research and Education (IDOR), Rio de Janeiro, Brazil

A large language model (LLM) can pass a medical examination. It cannot be called to the bedside at 3 a.m. to manage a patient in refractory shock, nor can it be held accountable for the consequences of a clinical decision. This distinction between producing a correct answer and exercising clinical judgement under professional responsibility lies at the heart of a growing challenge to postgraduate medical certification. LLMs have achieved passing-level performance on medical licensing examinations and increasingly impressive results on specialist board assessments [1,2,3]. This raises the question of whether correct answers reliably demonstrate the knowledge and judgement required for independent practice. Yet success in an examination, whether achieved by a physician or an LLM system, cannot establish clinical competence on its own. In intensive care medicine, where decisions must often be made rapidly, informed by incomplete and evolving information, and carry potentially irreversible consequences, this distinction matters. The question is what correct answers reveal about physicians and what further evidence justifies entrusting them with caring for critically ill patients.

European competency-based frameworks provide a common reference for defining the knowledge, skills and clinical reasoning expected of intensive care specialists [4, 5]. Their importance is particularly evident in Europe, where intensive care medicine is not universally recognised as an independent speciality and educational pathways vary considerably. In this diverse context, harmonised competency standards and structured assessment approaches can complement existing national qualifications, support consistency in evaluating professional competence and contribute to greater alignment of specialist training across Europe.

Maintaining credibility in the artificial intelligence (AI) era requires reassessing what certification should establish and how. Three priorities emerge: identifying the knowledge, reasoning and clinical judgement that physicians must demonstrate independently of AI; evaluating their ability to use AI critically, safely and responsibly without relinquishing professional judgement; and ensuring that examining bodies apply the same standards of validity, fairness, transparency and accountability to their own use of AI. The objective is not to diminish the importance of medical knowledge, but to ensure that certification continues to provide meaningful evidence of a physician’s readiness for independent clinical practice.

Validity concerns the interpretation and use of examination scores, rather than the questions themselves [6]. An LLM exceeding a predefined pass mark demonstrates performance on a particular set of assessment tasks, potentially influenced by prior exposure to similar content during training. Such performance cannot be extrapolated to competence in clinical practice. Similarly, LLMs’ ability to answer examination questions does not diminish the value of the knowledge those questions assess. In intensive care medicine, a solid foundation of knowledge remains essential for recognising clinical deterioration, interpreting physiological disturbances and initiating timely interventions. Physicians must have this knowledge readily accessible, without relying on external tools. Secure, supervised knowledge examinations therefore remain an essential component of certification, providing a standardised and reproducible assessment of the cognitive foundations of clinical practice. Their limitations reinforce the need to assess how candidates apply knowledge in clinical decisions.

In assessments conducted without direct supervision, including portfolios, case reports and reflective assignments, it is increasingly difficult to distinguish a candidate’s independent contribution from AI-generated or AI-assisted content. Prohibiting all AI use would be neither realistic nor educationally justified. Instead, assessment conditions should reflect the capabilities being evaluated. Where the objective is to establish independent knowledge and reasoning, external assistance must be excluded through appropriate supervision and examination safeguards. Where the critical use of AI forms part of the intended competence, its use should be explicitly authorised, transparently documented and evaluated against defined standards. The distinction is fundamental: certification must establish not only the quality of the final response, but also the extent to which the candidate can legitimately be attributed for the performance.

Emerging clinical evidence suggests that strong LLM performance does not necessarily translate into better physician decision-making. In a randomised trial, access to an LLM did not significantly improve physicians’ diagnostic reasoning, even though the model independently outperformed both study groups [7]. This discrepancy highlights an important limitation: the potential value of LLMs depends not only on the accuracy of their recommendations, but also on how clinicians interpret and integrate them into their decisions. Automation bias and the tendency to place excessive confidence in automated recommendations are recognised concerns [8]. In intensive care medicine, even a plausible and apparently well-supported recommendation may fail to account for evolving physiology, competing therapeutic priorities or individual patient circumstances. The ability to identify such limitations, critically appraise the proposed course of action and depart from it when clinically justified represents an increasingly important dimension of professional competence.

This evolving clinical context has direct implications for education and assessment. Beyond demonstrating independent clinical reasoning, physicians must develop the ability to evaluate LLM-generated recommendations, identify potentially consequential errors and determine when reliance on these tools is inappropriate. These capabilities require strong foundations in physiology, pharmacology and clinical reasoning. Educational approaches should therefore incorporate opportunities to confront plausible but inaccurate AI outputs, particularly in complex clinical scenarios where recommendations may appear convincing despite important omissions. Structured case discussions, simulation and supervised clinical encounters offer potential settings for developing and evaluating these skills. In certification, one possible approach would involve sequentially assessing the same clinical problem, first without AI assistance and then with an AI-generated recommendation containing a clinically significant error. Performance could be evaluated by identifying relevant clinical information, recognising the error, justifying the proposed management, and appropriately revising the decision. However, such approaches remain proposals rather than established assessment methods and would require rigorous evaluation of their validity, reliability, feasibility and fairness before implementation in high-stakes examinations [9, 10]. The key question is whether AI use contributes to safe, reasoned, and accountable decisions.

No single assessment method can adequately capture the complexity of intensive care practice. Standardised examinations provide a common reference for assessing knowledge and clinical reasoning, while longitudinal workplace-based assessment offers complementary evidence of how these capabilities are applied in clinical practice. Programmatic assessment integrates multiple sources of evidence, including direct observation, structured feedback and formal examinations, collected across different clinical contexts and over time [11, 12]. Within this framework, entrustable professional activities (EPAs) support the transition from demonstrated competence to decisions about the level of supervision required for specific clinical responsibilities [13, 14]. Integrating standardised assessment with longitudinal evidence of clinical performance may therefore provide a more comprehensive basis for evaluating readiness for independent practice. However, workplace-based assessment presents challenges, including variability between assessors, limited opportunities for direct observation and potential bias, highlighting the importance of structured implementation, assessor training and systematic evidence collection. Formal certification and longitudinal assessment are complementary measures of competence and clinical autonomy.

The implications of AI also extend to examining bodies themselves. AI applications are increasingly being considered for question development, quality assurance, examination monitoring and candidate feedback. Although these tools may improve efficiency, their adoption must be justified by more than operational convenience. Their intended purpose, limitations and influence on assessment outcomes should be clearly documented, with appropriate validation, protection of confidential examination materials and candidate data, and ongoing evaluation of accuracy and fairness. Attention is required when AI contributes to scoring, monitoring or decisions that may affect candidate progression. The European Union Artificial Intelligence Act identifies certain AI systems used to evaluate learning outcomes or monitor prohibited behaviour during examinations as high-risk and establishes specific regulatory obligations [15]. Compliance, however, cannot substitute for professional oversight. Examining bodies must retain responsibility for the integrity of their assessments, ensure that consequential decisions remain subject to meaningful human review and provide transparent mechanisms for challenge and appeal.

## Conclusion

The defining challenge of the AI era is not whether machines can pass medical examinations, but whether our education and certification systems can adequately distinguish correct answers from sound clinical judgement and demonstrated knowledge from the ability to act on it responsibly. AI does not diminish the value of medical knowledge or render established examinations obsolete. Rather, it challenges us to strengthen the evidence on which we establish professional competence. In intensive care medicine, where clinical decisions carry profound consequences, certification must represent more than examination success: it must provide credible evidence that physicians can translate knowledge into decisions, recognise the limits of their own judgement and remain accountable for their actions. Passing an examination demonstrates achievement of a standard; entrusting a physician with the care of critically ill patients demands evidence beyond that.
